# Supplementary material for: Omadacycline, Eravacycline, and Tigecycline Express Anti-Mycobacterium abscessus Activity In Vitro
Source: Microbiol Spectr. 2023 May 4;11(3):e00718-23. doi: 10.1128/spectrum.00718-23 (PMC10269442; doi:10.1128/spectrum.00718-23)
Supplement: Supplemental file 1 — Supplemental material. Download spectrum.00718-23-s0001.pdf, PDF file, 0.5 MB [file spectrum.00718-23-s0001.pdf]

Table S1. MICs of 4 tetracycline-analogs for 193 clinical *M. abscessus* isolates incubated at 30°C

Isolates	Subspecies	MIC (μg/mL)			
		Omadacycline	Eravacycline	Tigecycline	Sarecycline
A8	*abscessus*	0.5	0.063	0.25	>8
3	*abscessus*	0.25	0.125	0.063	>8
A213	*abscessus*	0.5	0.125	0.125	>8
A337	*abscessus*	0.5	0.125	0.125	>8
G70	*abscessus*	0.5	0.125	0.125	>8
G75	*massiliense*	0.5	0.25	0.125	>8
A297	*abscessus*	1	0.125	0.125	>8
G149	*abscessus*	1	0.125	0.125	>8
G87	*massiliense*	1	0.125	0.125	>8
A182	*abscessus*	1	0.25	0.125	4
G90	*abscessus*	0.5	0.25	0.25	4
A176	*abscessus*	0.5	0.25	0.25	>8
A305	*abscessus*	0.5	0.25	0.25	>8
A232	*abscessus*	0.5	0.25	0.25	>8
A244	*abscessus*	0.5	0.25	0.25	>8
A266	*abscessus*	0.5	0.25	0.25	>8
A228	*massiliense*	0.5	0.25	0.25	>8
A317	*abscessus*	0.5	0.25	0.25	>8
A254	*massiliense*	0.5	0.25	0.25	>8
G124	*massiliense*	0.5	0.125	0.25	>8
G72	*abscessus*	0.5	0.25	0.25	>8
A217	*abscessus*	1	0.25	0.25	>8
A249	*abscessus*	1	0.25	0.25	>8
A312	*abscessus*	1	0.25	0.25	>8
129	*abscessus*	1	0.25	0.25	>8
G129	*abscessus*	1	0.25	0.25	>8
G179	*abscessus*	1	0.25	0.25	>8
G219	*abscessus*	0.25	0.25	0.5	>8
A54	*abscessus*	2	0.25	0.5	>8
G143	*abscessus*	0.5	0.25	0.5	>8
G172	*abscessus*	0.5	0.25	0.5	>8
A59	*abscessus*	1	0.25	0.5	>8
A73	*abscessus*	1	0.25	0.5	>8
A79	*abscessus*	1	0.25	0.5	>8
A215	*abscessus*	1	0.25	0.5	>8
A321	*abscessus*	1	0.25	0.5	>8
G161	*abscessus*	1	0.25	0.5	>8

G165	*abscessus*	1	0.25	0.5	>8
G169	*abscessus*	1	0.25	0.5	>8
A295	*massiliense*	1	0.25	0.25	>8
G175	*abscessus*	1	0.25	0.5	>8
G74	*massiliense*	1	0.25	0.25	>8
G77	*massiliense*	1	0.25	0.25	>8
G198	*abscessus*	1	0.25	0.5	>8
A274	*abscessus*	2	0.25	0.5	>8
G142	*abscessus*	0.5	0.5	0.25	>8
A329	*abscessus*	0.5	0.5	0.25	>8
G215	*abscessus*	0.5	0.5	0.5	>8
G151	*abscessus*	1	0.5	0.5	>8
G163	*abscessus*	0.5	0.5	0.5	>8
G101	*massiliense*	1	0.25	0.25	>8
A323	*massiliense*	2	0.25	0.25	>8
G177	*abscessus*	0.5	0.5	0.5	>8
G108	*massiliense*	2	0.25	0.25	>8
G141	*massiliense*	0.5	0.25	0.5	8
A69	*abscessus*	1	0.5	0.5	>8
A137	*abscessus*	1	0.5	0.5	>8
A222	*massiliense*	1	0.5	0.5	>8
A183	*abscessus*	1	0.5	0.5	>8
A289	*massiliense*	1	0.5	0.5	>8
A218	*abscessus*	1	0.5	0.5	>8
A350	*abscessus*	1	0.5	0.5	>8
G88	*massiliense*	1	0.25	0.5	>8
G73	*abscessus*	1	0.5	0.5	>8
289	*abscessus*	1	0.5	0.5	>8
G113	*abscessus*	1	0.5	0.5	>8
G139	*abscessus*	1	0.5	0.5	>8
G150	*abscessus*	1	0.5	0.5	>8
G164	*abscessus*	1	0.5	0.5	>8
G176	*abscessus*	1	0.5	0.5	>8
A330	*abscessus*	2	0.5	0.5	>8
G99	*abscessus*	2	0.5	0.5	>8
G106	*abscessus*	2	0.5	0.5	>8
G120	*abscessus*	2	0.5	0.5	>8
G121	*abscessus*	2	0.5	0.5	>8
G122	*abscessus*	2	0.5	0.5	>8
G125	*abscessus*	2	0.5	0.5	>8
G199	*massiliense*	1	0.5	0.5	>8

G128	*abscessus*	2	0.5	0.5	>8
G145	*abscessus*	2	0.5	0.5	>8
G148	*abscessus*	2	0.5	0.5	>8
G152	*abscessus*	2	0.5	0.5	>8
G220	*massiliense*	1	0.25	0.5	>8
G82	*abscessus*	4	0.5	0.5	>8
G201	*abscessus*	1	0.5	1	>8
A38	*abscessus*	1	0.5	1	>8
G134	*abscessus*	1	0.5	1	>8
G98	*massiliense*	1	2	1	>8
G216	*abscessus*	1	0.5	1	>8
G118	*abscessus*	2	0.5	1	>8
G110	*massiliense*	1	0.5	1	>8
A35	*abscessus*	1	0.5	1	>8
A315	*abscessus*	1	0.5	1	>8
G173	*massiliense*	1	0.5	1	>8
G78	*abscessus*	1	0.5	1	>8
G89	*abscessus*	1	0.5	1	>8
A243	*abscessus*	2	0.5	1	>8
G76	*abscessus*	2	0.5	1	>8
G104	*abscessus*	2	0.5	1	>8
G188	*massiliense*	1	1	1	>8
G109	*abscessus*	1	0.5	1	>8
G207	*massiliense*	1	0.5	1	>8
G159	*abscessus*	1	0.5	1	>8
G178	*abscessus*	1	0.5	1	>8
G192	*abscessus*	1	0.5	1	>8
G147	*massiliense*	2	1	1	>8
G200	*abscessus*	1	0.5	1	>8
G105	*massiliense*	4	1	1	>8
G107	*massiliense*	4	1	1	>8
A58	*abscessus*	2	0.5	1	>8
A175	*abscessus*	2	0.5	1	>8
G126	*massiliense*	4	1	1	>8
G114	*abscessus*	2	0.5	1	>8
G180	*abscessus*	2	0.5	1	>8
G181	*abscessus*	2	0.5	1	>8
G185	*abscessus*	2	0.5	1	>8
G187	*abscessus*	2	0.5	1	>8
G194	*abscessus*	2	0.5	1	>8
G208	*abscessus*	2	0.5	1	>8

G195	*abscessus*	2	0.5	2	>8
G170	*abscessus*	4	0.5	2	>8
G135	*massiliense*	4	1	1	>8
G197	*abscessus*	0.5	1	0.5	4
G102	*abscessus*	2	1	0.5	>8
G138	*massiliense*	4	1	1	>8
G93	*abscessus*	1	1	1	>8
G146	*abscessus*	1	1	1	>8
G144	*abscessus*	1	1	1	>8
G211	*abscessus*	1	1	1	>8
G117	*abscessus*	2	1	1	>8
G119	*abscessus*	2	1	1	>8
G136	*abscessus*	2	1	1	>8
G162	*abscessus*	2	1	1	>8
G206	*abscessus*	2	1	1	>8
A126	*abscessus*	2	1	2	>8
G123	*abscessus*	2	1	2	>8
G103	*abscessus*	1	1	2	>8
A173	*massiliense*	2	1	2	>8
A197	*abscessus*	2	1	2	>8
A186	*massiliense*	2	1	2	>8
A255	*abscessus*	2	1	2	>8
A311	*abscessus*	2	1	2	>8
A247	*massiliense*	2	1	2	>8
G84	*abscessus*	2	1	2	>8
A267	*massiliense*	2	2	2	>8
A268	*massiliense*	2	1	2	>8
G86	*abscessus*	2	1	2	>8
G94	*abscessus*	2	1	2	>8
G115	*abscessus*	2	1	2	>8
G133	*abscessus*	2	1	2	>8
G155	*abscessus*	2	1	2	>8
G160	*abscessus*	2	1	2	>8
G174	*abscessus*	2	1	2	>8
G184	*abscessus*	2	1	2	>8
G186	*abscessus*	2	1	2	>8
G204	*abscessus*	2	1	2	>8
G116	*massiliense*	2	2	2	>8
G205	*abscessus*	2	1	2	>8
G210	*abscessus*	2	1	2	>8
G213	*abscessus*	2	1	2	>8

G158	*abscessus*	4	1	2	>8
G193	*abscessus*	1	2	1	>8
G196	*abscessus*	1	2	1	>8
A40	*abscessus*	2	2	2	>8
A25	*abscessus*	4	2	4	>8
A51	*abscessus*	2	2	4	>8
A233	*abscessus*	2	2	4	>8
G127	*abscessus*	2	2	4	>8
G137	*massiliense*	2	1	2	>8
G218	*abscessus*	2	2	4	>8
A10	*abscessus*	4	2	4	>8
G156	*massiliense*	2	1	2	>8
A189	*abscessus*	4	2	4	>8
G79	*abscessus*	4	2	4	>8
2	*abscessus*	4	2	4	>8
G157	*massiliense*	2	1	2	>8
G91	*abscessus*	4	2	4	>8
G189	*massiliense*	2	1	2	>8
G153	*massiliense*	2	1	4	>8
G111	*abscessus*	4	2	4	>8
G112	*abscessus*	4	2	4	>8
A205	*massiliense*	4	2	4	>8
G132	*abscessus*	4	2	4	>8
G85	*massiliense*	4	2	4	>8
G190	*massiliense*	4	2	4	>8
G140	*abscessus*	4	2	4	>8
G182	*abscessus*	4	2	4	>8
G183	*abscessus*	2	4	1	>8
A49	*abscessus*	4	4	4	>8
G203	*massiliense*	4	2	4	>8
A39	*massiliense*	4	4	8	>8
A63	*massiliense*	8	4	8	>8
G95	*massiliense*	8	4	8	>8

Table S2. MBC/MIC ratios of omadacycline, eravacycline and tigecycline for *M. abscessus* reference strains and clinical isolates incubated at 37°C and 30°C[a]

Isolates[b]	Subspecies	MBC/MIC ratios		
		Omadacycline	Eravacycline	Tigecycline
ATCC 19977	*abscessus*	>8	>8	>8
CIP 108297	*massiliense*	>8	>8	>8
A175	*abscessus*	>8	>8	>8
A182	*abscessus*	>8	>8	>4
A205	*massiliense*	>8	>8	>8
A58	*abscessus*	>8	>8	>8
A8	*abscessus*	>8	>8	>8
A197	*abscessus*	>8	>8	>8
A183	*abscessus*	>8	>8	>8
A79	*abscessus*	>8	>8	>8
A59	*abscessus*	>8	>8	>8
A73	*abscessus*	>8	>8	>8
A137	*abscessus*	>8	>8	>8
A69	*abscessus*	>8	>8	>8
289	*abscessus*	>8	>8	>8
G101	*massiliense*	>8	>8	>8
G102	*abscessus*	>8	>8	>8
G104	*abscessus*	>8	>8	>8
G105	*massiliense*	>8	>8	>8
G106	*abscessus*	>8	>8	>8

[a]An MBC/MIC ratio >4 indicates that the agent is bacteriostatic; values >8 indicate that the MBC is greater than the highest concentration tested. MBC/MIC ratios determined for organisms incubated at 37°C and 30°C were the same.

[b]Subsp. *abscessus* ATCC 19977 and subsp. *massiliense* CIP 108297 were reference strains.

Table S3. Comparison of the MICs of 4 tetracycline analogs for 29 *M. abscessus* isolates cultured at 37°C versus 30°C

Isolates	Subspecies	MIC (μg/ml)							
		OMC[a]		ERC[a]		TGC[a]		SAC[a]	
		37°C	30°C	37°C	30°C	37°C	30°C	37°C	30°C
A8	*abscessus*	0.5	0.5	0.25	0.25	0.125	0.063	>8	>8
A337	*abscessus*	0.5	0.5	0.25	0.125	0.25	0.125	>8	>8
A49	*abscessus*	1	4	0.5	4	0.5	4	>8	>8
A182	*abscessus*	1	1	1	0.125	0.5	0.25	>8	>8
A249	*abscessus*	1	1	0.25	0.25	0.25	0.25	>8	>8
A297	*abscessus*	1	1	0.25	0.125	0.25	0.125	>8	>8
A350	*abscessus*	1	1	0.5	0.5	0.5	0.5	>8	>8
G73	*abscessus*	1	1	0.5	0.5	0.5	0.5	>8	>8
G74	*massiliense*	1	1	0.25	0.25	0.25	0.25	>8	>8
G88	*massiliense*	1	1	1	0.5	0.5	0.25	>8	>8
G142	*abscessus*	1	0.5	1	0.25	0.5	0.5	>8	>8
A255	*abscessus*	2	2	2	2	1	1	>8	>8
A274	*abscessus*	2	2	0.5	0.5	0.5	0.25	>8	>8
A312	*abscessus*	2	1	0.5	0.25	0.5	0.25	>8	>8
A323	*massiliense*	2	2	1	0.25	1	0.25	>8	>8
A329	*abscessus*	2	0.5	0.5	0.25	0.5	0.5	>8	>8
A330	*abscessus*	2	2	0.5	0.5	0.5	0.5	>8	>8
129	*abscessus*	2	1	0.5	0.25	0.5	0.25	>8	>8
2	*abscessus*	2	4	2	4	2	2	>8	>8
G103	*abscessus*	2	1	2	2	2	1	>8	>8
G116	*massiliense*	2	2	2	2	2	2	>8	>8
A58	*abscessus*	4	2	1	1	2	0.5	>8	>8
A63	*massiliense*	4	8	8	8	8	4	>8	>8
A175	*abscessus*	4	2	2	1	2	0.5	>8	>8
G94	*abscessus*	4	2	4	2	2	1	>8	>8
G115	*abscessus*	4	2	4	2	2	1	>8	>8
G132	*abscessus*	4	4	4	4	4	2	>8	>8
A197	*abscessus*	8	2	8	2	4	1	>8	>8
A205	*massiliense*	8	4	2	4	2	2	>8	>8

[a]Abbreviations: OMC = omadacycline; ERC = eravacycline; TGC = tigecycline; SAC = sarecycline.

Table S4. Comparison of MICs of omadacycline, eravacycline, tigecycline and sarecycline for *M. abscessus* reference strains determined in 6 studies

Study (Year & Country)	Incubation temp/time	Reference strain	MIC (μg/mL)			
			OMC[a]	ERC[a]	TGC[a]	SAC[a]
Bax *et al.* (21) (2019, The Netherlands)	35°C/3-5d	CIP 104536	4	-	4	-
Kaushik *et al.* (14) (2019, USA)	30°C/3d	ATCC 19977	1	0.5	1	-
Brown-Elliott *et al.* (17) (2020, USA)	30°C/3d	ATCC 19977	0.5	-	0.12-0.5	-
Nicklas *et al.* (15) (2022, USA)	30°C/3d	ATCC 19977	0.375	-	0.125	-
Zhang *et al.* (20) (2022, China)	37°C/3d	ATCC 19977	0.25	0.0625	0.25	>8
Current study (2023, China)	30°C/3d	ATCC 19977	2	1	0.5	>8
	37°C/3d	ATCC 19977	1	0.5	0.5	>8
	30°C/3d	CIP 108297	1	1	0.5	>8
	37°C/3d	CIP 108297	2	1	1	>8

[a]Abbreviations: OMC, omadacycline; ERC, eravacycline; TGC, tigecycline; SAC, sarecycline.
